# A Mediterranean mesophotic coral reef built by non-symbiotic scleractinians

**DOI:** 10.1038/s41598-019-40284-4

**Published:** 2019-03-05

**Authors:** Giuseppe Corriero, Cataldo Pierri, Maria Mercurio, Carlotta Nonnis Marzano, Senem Onen Tarantini, Maria Flavia Gravina, Stefania Lisco, Massimo Moretti, Francesco De Giosa, Eliana Valenzano, Adriana Giangrande, Maria Mastrodonato, Caterina Longo, Frine Cardone

**Affiliations:** 1 Dipartimento di Biologia, Università degli Studi di Bari Aldo Moro, Via Orabona 4, 70125 Bari, Italy; 2 Consorzio Nazionale Interuniversitario per le Scienze del Mare (CoNISMa), Piazzale Flaminio 9, 00196 Roma, Italy; 3 Istituto di Ricerca sugli Ecosistemi Terrestri (CNR-IRET), Via Salaria km. 29.300 - 00015 Monterotondo Scalo, Roma, Italy; 4 Dipartimento di Biologia, Università di Roma “Tor Vergata”, Via della Ricerca Scientifica s.n.c, 00133 Roma, Italy; 5 Dipartimento di Scienze della Terra e Geoambientali, Università degli Studi di Bari Aldo Moro, Via Orabona 4, 70124 Bari, Italy; 6 Environmental Surveys S.r.l. (ENSU), Via de Gasperi, 74123 Taranto, Italy; 7 Dipartimento di Scienze e Tecnologie Biologiche ed Ambientali, Università del Salento. Via Provinciale Lecce-Monteroni, 73100 Lecce, Italy

## Abstract

This is the first description of a Mediterranean mesophotic coral reef. The bioconstruction extended for 2.5 km along the Italian Adriatic coast in the bathymetric range −30/−55 m. It appeared as a framework of coral blocks mostly built by two scleractinians, *Phyllangia americana mouchezii* (Lacaze-Duthiers, 1897) and *Polycyathus muellerae* (Abel, 1959), which were able to edify a secondary substrate with high structural complexity. Scleractinian corallites were cemented by calcified polychaete tubes and organized into an interlocking meshwork that provided the reef stiffness. Aggregates of several individuals of the bivalve *Neopycnodonte cochlear* (Poli, 1795) contributed to the compactness of the structure. The species composition of the benthic community showed a marked similarity with those described for Mediterranean coralligenous communities and it appeared to be dominated by invertebrates, while calcareous algae, which are usually considered the main coralligenous reef-builders, were poorly represented. Overall, the studied reef can be considered a unique environment, to be included in the wide and diversified category of Mediterranean bioconstructions. The main reef-building scleractinians lacked algal symbionts, suggesting that heterotrophy had a major role in the metabolic processes that supported the production of calcium carbonate. The large amount of available suspended organic matter in the area could be the main nutritional source for these species, as already suggested in the literature referred to Mediterranean cold-water corals.

## Introduction

The most important marine bioconstructions are coral reefs, which are well known as biodiversity hot spots^1,2^. Coral reefs mainly occur in the oligotrophic waters of the western Atlantic and Indo-Pacific regions, within the latitude of 30°N and 30°S^3,4^. They are mainly composed of stony corals, helped in the bioconstruction by several species of invertebrates with carbonate skeletons and coralline algae. Coral reefs form through successive stages of growth involving the deposition and consolidation of the remains of these reef-building benthic organisms^5^, being the carbonate deposition, typical of these habitats, enhanced by the close mutualistic symbiosis of corals with microalgae^6,7^.

Therefore, the vertical distribution of a reef is primarily affected by light availability^8^, even though coral reefs that live close to the surface are only a small portion of the complete coral reef ecosystem. Indeed, it is now known that mesophotic coral reefs (MCRs) are widespread and diversified worldwide. They are found at depths ranging from 30–40 m and extending to the limit of the photic zone, which may be up to 200 m in adequate environmental conditions^9,10^. MCRs are usually built by light-dependent corals whose photosynthetic symbionts tolerate middle- to low-light conditions^11,12^. Other taxa that provide structural habitat, include sponge and algal species^13,14^. Compared with their shallow-water counterparts, less is known about this ecosystem, which is mainly widespread beyond the range where diving operations are safe.

In Mediterranean Sea, coral reefs were widely distributed in the past^15,16^, but they currently have a reduced extension and distribution. Reefs built by the scleractinian *Cladocora caespitosa* (Linnaeus, 1767) have been occasionally reported in shallow waters^16^, whereas in deeper dark environments, *Desmophyllum pertusum* (=*Lophelia pertusa* (Linnaeus, 1758)) and *Madrepora oculata* Linnaeus, 1758 may form large three-dimensional (3D) carbonate structures^17,18^. Moreover, some scleractinian species contribute to the coralligenous bioconstruction, which is the main calcareous formation of biogenic origin of the Mediterranean^19^. According to the literature, calcareous algae are in fact the basic bioconstructors of coralligenous formations^20–22^. However, associated sessile invertebrates with calcified skeletons (i.e., mainly cnidarians, annelids and bryozoans) also contribute to the bioconstruction, as they increase and consolidate the carbonate structure, sometimes becoming more abundant than the encrusting algae; thus, coralligenous can be considered a mosaic of different communities^19^. The contribution of different taxa depends on parameters such as depth, morphology and geographic features of the site^23–28^. Substrate topography, light and water temperature strongly affect the nature of the outcrops, leading to the edification of banks on the horizontal seafloor and cliffs on inclined to sub-vertical slopes^29,30^. In Mediterranean Sea, coralligenous concretions cover an area of at least 2,763 km^2^^31^, and they are typically distributed between 20 and 100 m of depth. Along the Apulian continental shelf (e.g. South Adriatic and northern Ionian Sea), coralligenous extends over approximately 450 km^2^, likely representing the most relevant habitat in this area^31^.

By coupling marine biology and geology approaches, in this paper we describe the main features of the mesophotic bioconstruction present along the Adriatic Apulian coast. The goals of this study were to provide the small-scale spatial distribution, the census of the main builder species, and the list of the associated benthic species of such undescribed Mediterranean bioconstruction.

## Results

### Morphology and lithological features of the substrate

The analysis of the high-resolution side-scan sonar (SSS) and sub-bottom profiler (SBP) datasets was used to describe the main morphological and lithological features of the investigated marine area. This area was dominated by an irregular EW oriented slope that connects a flat upper surface (depths of approximately 30–35 m) to deeper environments (depths of approximately 45–50 m).

**Figure 1:**
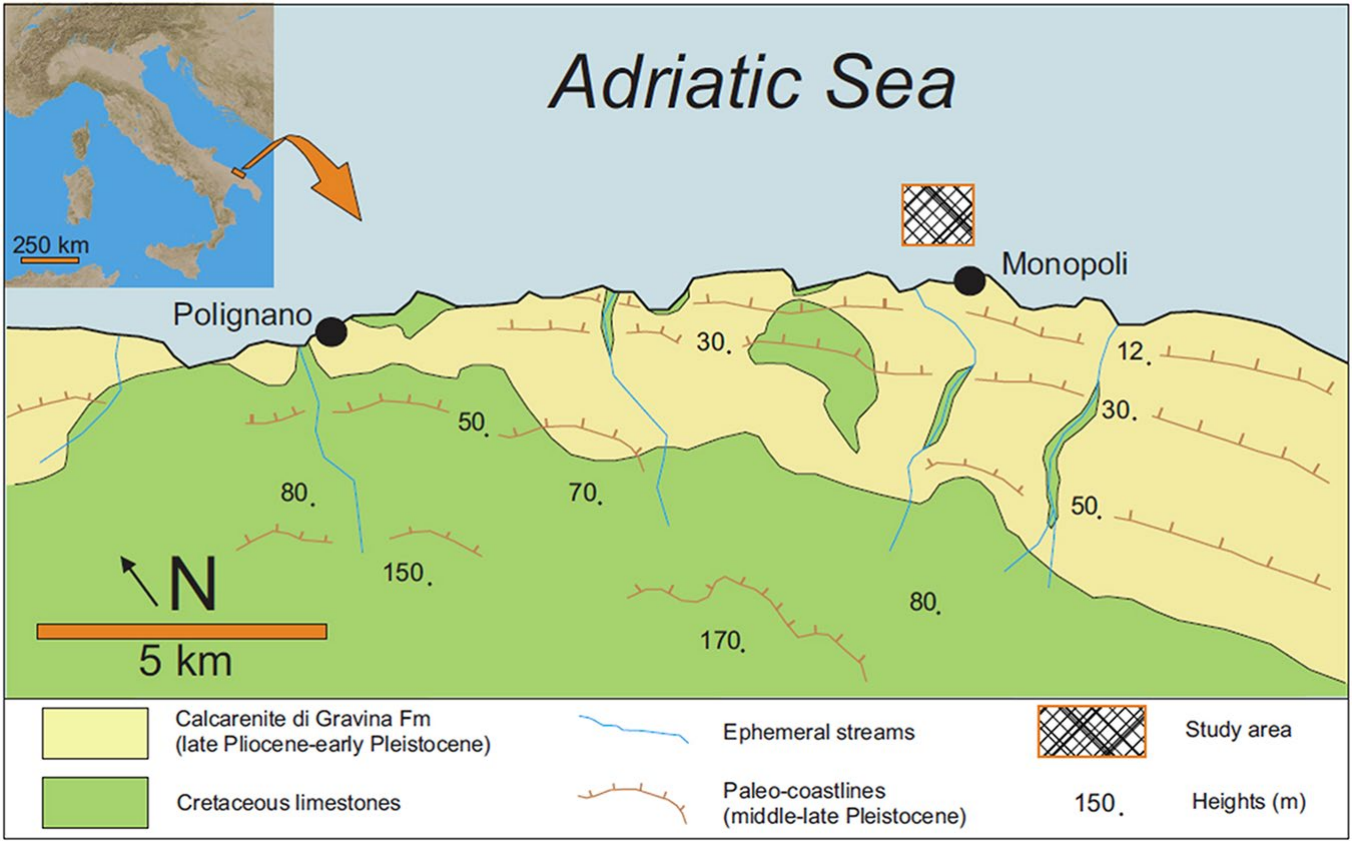
The study area. Location of the study area and schematic morphological and geological setting of the Monopoli area.

**Figure 2:**
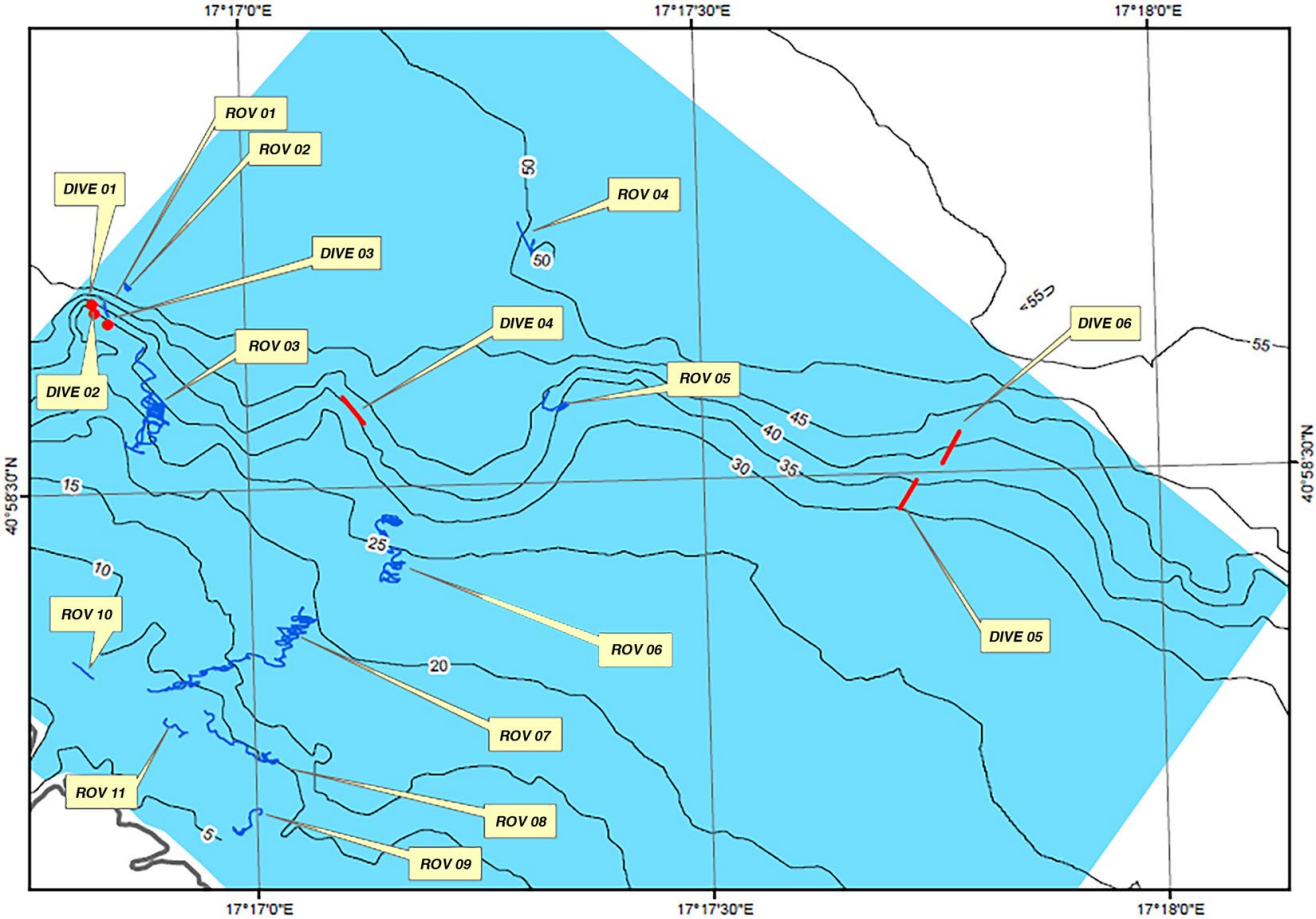
Map of the study area with the localization of navigation tracks and dives. Bathymetric map of the study area with remotely operated vehicle navigation tracks (blue) and scuba-diving transects (red).

**Figure 3:**
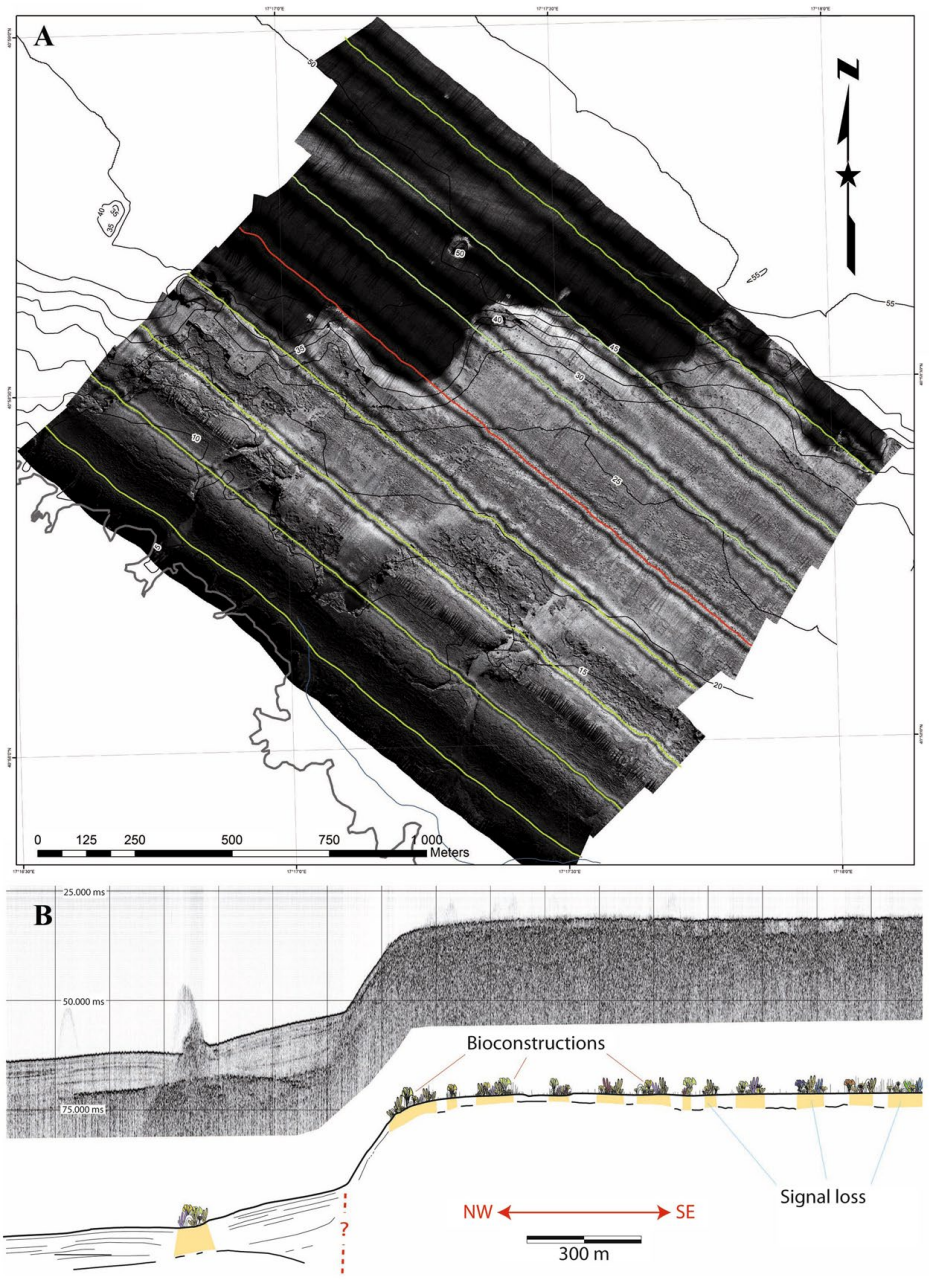
Geophysical mapping results. (**A**) Side-scan sonar map. Note the presence of channelized erosional forms in the southwestern sector, the complex morphology of the EW-oriented slope, and the blackish deeper sector below the base of the slope. (**B**) Sub-bottom profiler section across the study area (location shown in red in **A**). Note that the distribution of bioconstructions on the sea bottom can be detected as signal loss sectors. The general morphology of the area is dominated by the presence of an EW slope, which is probably related to a fault with a similar orientation (red dotted line).

The recorded structure was located along the EW fault and was approximately perpendicular to the coastline. This morphotectonic element can be recognized by looking at the bathymetric lines (Figs 1, 2) and is well visible on the SSS map (Fig. 3A). A high acoustic impedance bedrock bottom occurs in the upper flat sector, while poorly consolidated sedimentary deposits overlay the bedrock in the deeper part of the system. These deposits are approximately 5 m thick and consist of alternations of different lithologies and/or sediments with a variable degree of consolidation; these deposits likely represent the result of one of the latest Quaternary sedimentation phases. The presence of current bioconstructions in the shallower area and, only locally, in the deeper sectors is suggested by the occurrence of localized areas of signal loss^32,33^ (Fig. 3A, B). The SSS dataset allows for the description of the main areal features of the same marine sector.

Main linear features are recognizable in shallow marine environments and are related with channel-like erosional morphologies that are incised in the bedrock; these features represent the prosecution of the main present-day ephemeral streams. Different sedimentary substrates were observed in the littoral (i.e., from the coastline up to depths of 30–35 m) and deep areas (i.e., from the base of the slope up to depths of 50 m). In the deepest sectors, the seabed was generally monotonous and represented by fine-graded sediments. In the shallower zones, the seabed consisted of different lithologies related to the presence of the bedrock, coarse- or fine-grained loose sediments, and various types of biological coverage on soft and hard substrates. The SSS and SBP databases define the seabed classification, and Supplementary Table S1 summarizes the geophysical criteria used for the classification and shows sample images for each of the seven classes.

### Biotope mapping of the study area

**Figure 4:**
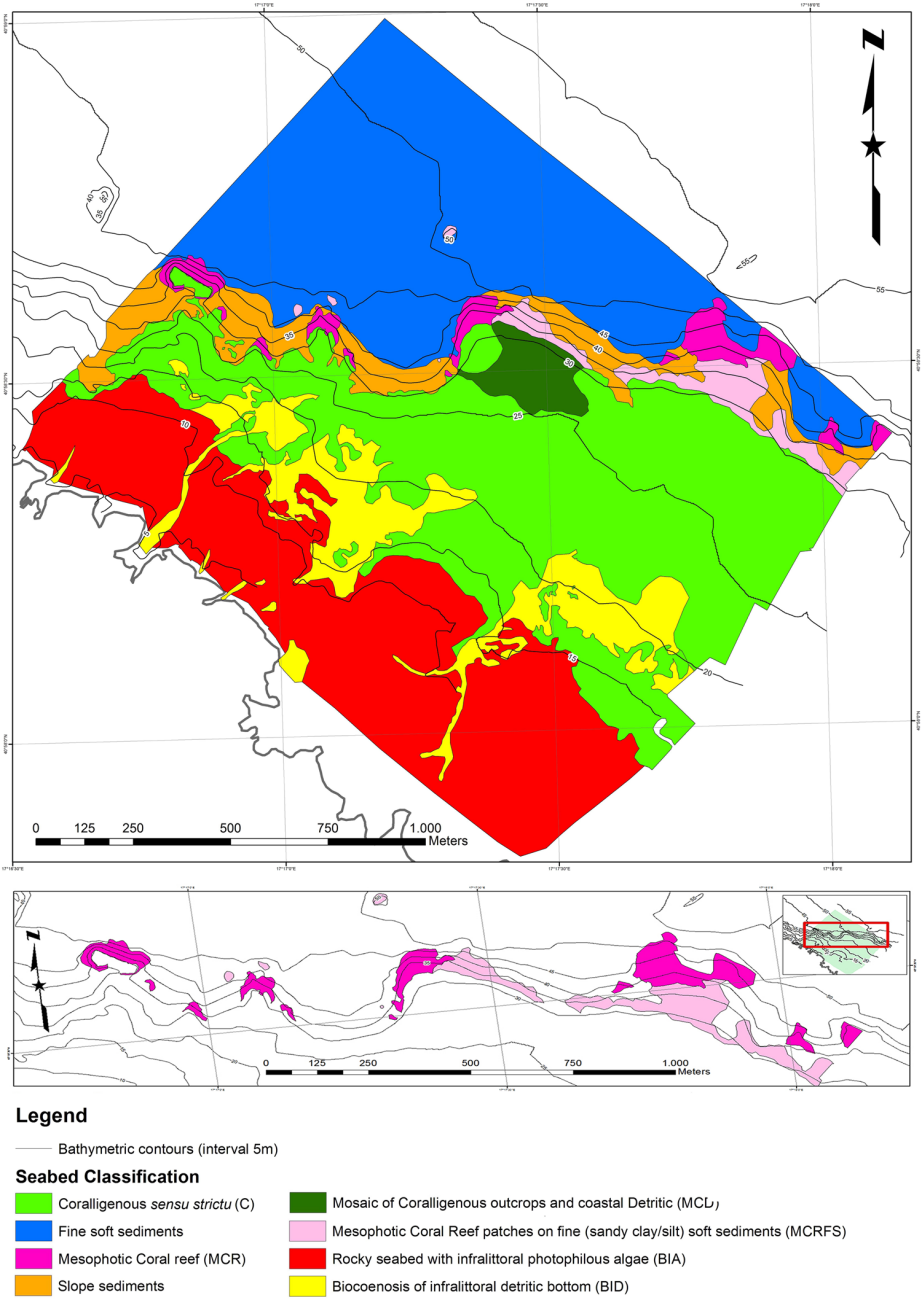
Map of the study area produced using geophysical and bionomic data, seabed video, scuba dives and sample analysis with the detail of the mesophotic coral reef distribution.

**Figure 5:**
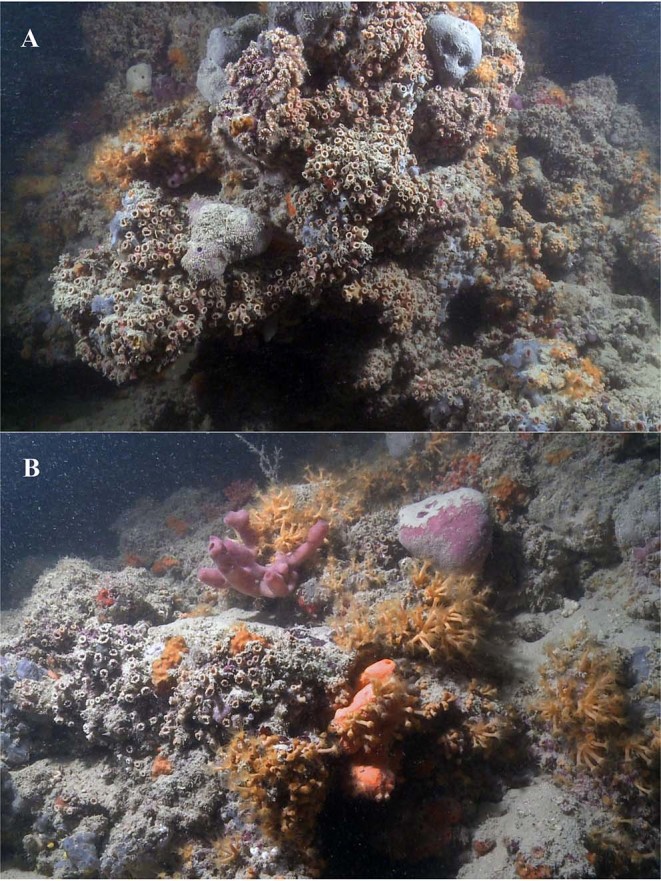
*In situ* images of the mesophotic coral reef. (**A**) Sub-vertically and (**B**) horizontally oriented, both showing heavy siltation over the reef structure.

The final classification map shown in Fig. 4 was obtained using data from geophysical surveys, remotely operated vehicle (ROV) profiles, scuba diving, and sampling surveys. In particular, the map resulted from a comparison between the geophysical and bionomic facies (see Supplementary Tables S1, S2 for details); thus, the map included mixed lithological and biological criteria. The MCR (Figs 4, 5) was discontinuously detected along 2.09 km of coastline within a bathymetric range of 30–55 m, covering a total of 0.050 km^2^. At the same bathymetric range, an additional surface of 0.048 km^2^ consisted of MCR patches on fine (sandy clay/silt) soft sediments (MCRFS) (Fig. 4). Both MCR and MCRFS fall into a quadrilateral, whose vertices are A = 17° 17.216, 40° 58.993; B = 17° 18.124, 40° 58.4; C = 17° 17.617, 40° 57.907; and D = 17° 16.644, 40° 58.532. It appeared as a steep slope rising from a muddy soft bottom up to a depth of approximately 30 m. At its upper limit, the coral framework was replaced by foliaceous Peyssonneliaceae algae, which together with encrusting and erected bryozoans, assumed the roles of the main engineers. Other suspension feeders, such as demosponges and large colonies of the scleractinian *Cladocora caespitosa* were widely represented. At a depth of approximately 25 m, coralligenous outcrops had a high structural complexity, reaching a thickness of approximately 5 m. They were usually mixed with coarse terrigenous and organogenic incoherent substrates that alternated with small patches of *Posidonia oceanica* tufts. Between depths of 10 and 15 m, photophilous algae were dominant and mixed with small and scattered 10–15 cm thick coralligenous bioconstructions that consisted mainly of brown algae and massive sponges. From the coastline to a depth of 10 m, the seabed consisted of a plateau covered by coarse sand and infralittoral photophilous algae dominated by frondose coralline and tiny brown algae with small occasional patches of fine sand (Fig. 4).

### Reef building and structuring taxa

**Figure 6:**
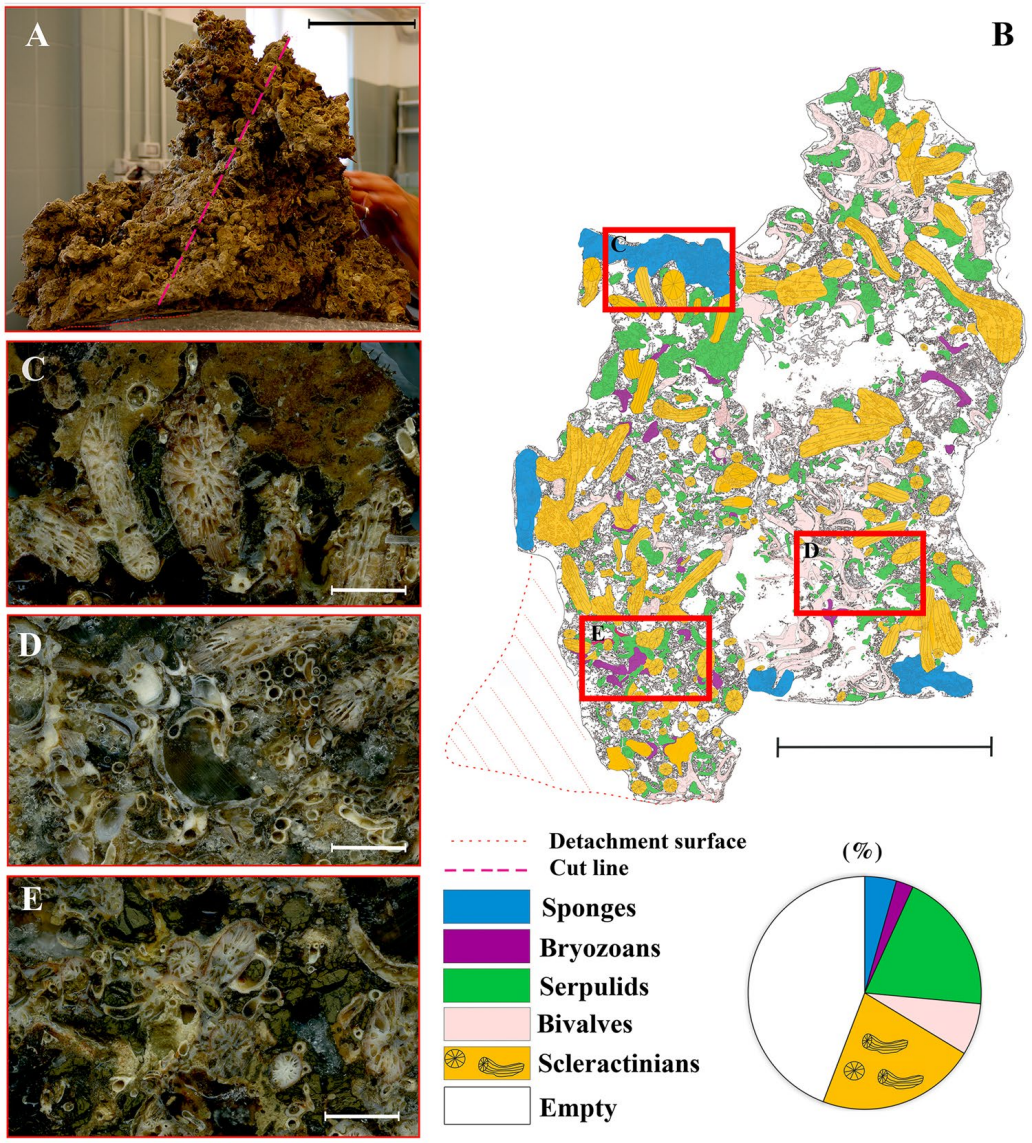
Coral reef block with details of the main builder taxa identified. (**A**) Sampled block with indication of cut line (red dotted line). (**B**) Schematic representation of the cutting surface. Red boxes indicate the different positions where pictures C ÷ E were taken within the block; (**C**) external surface with scleractinian corals and sponges; (**D**) intermediate portion mainly characterized by serpulid tubes and bivalves; (**E**) basal portion with bryozoans, serpulids and eroded scleractinian skeletons. Scale bars: A, B = 10 cm; C ÷ E = 1 cm.

**Figure 7:**
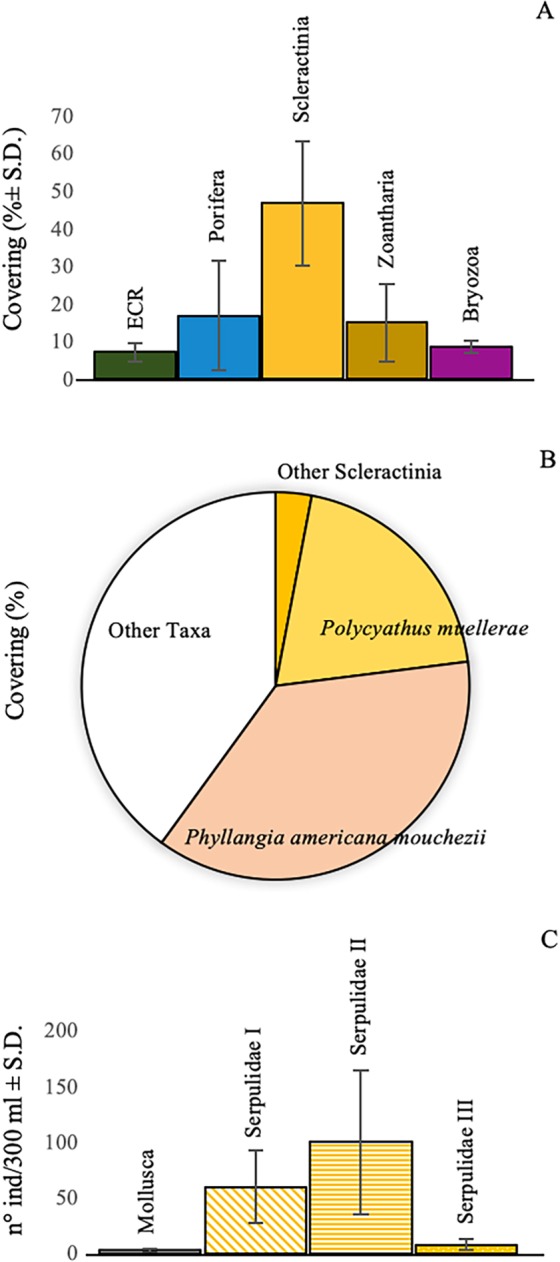
Contribution of the main taxa to the mesophotic coral reef. Covering values in percentage of the main structuring taxa. The values were obtained from image analysis of: (**A**) field photo/videos (ECR = Encrusting Coralline Rhodophytes); (**B**) taxonomic sample analysis. In (**C**), for molluscs and serpulids, abundance values refer to n° of individuals in 300 ml of bioconstruction (I, II, III = serpulids’ size classes).

**Figure 8:**
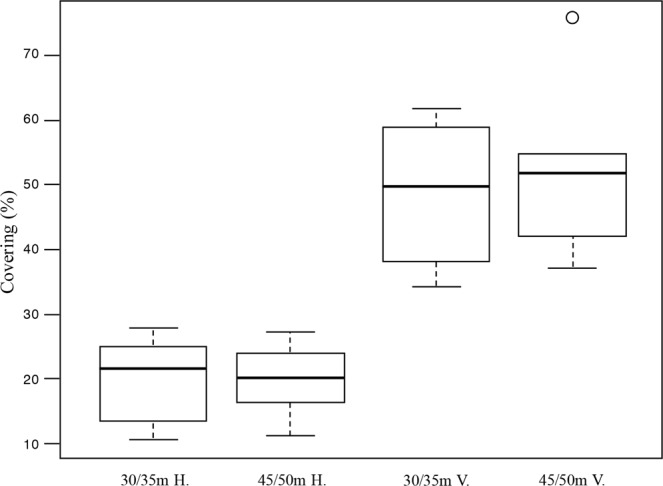
Box plots of scleractinian covering (%) related to the substratum inclination: Horizontal (H.) vs Vertical (V.) and depth (30 ÷ 35 vs 45 ÷ 50 m).

According to the analysis of the sampled material and video images, the reef appeared as a continuous framework of coral blocks. It showed a marked development in terms of thickness: the largest blocks that were sampled reached approximately 0.4 m (Fig. 6A), although the field surveys indicated values up to 2 m. The reef was mainly built by two scleractinian species, *Phyllangia americana mouchezii* (Lacaze-Duthiers, 1897) and *Polycyathus muellerae* (Abel, 1959), which can both edify a secondary substrate of high structural complexity that provides heterogeneous microhabitats (Fig. 5A, B). Their colonies were present in 100% of the analysed images, but the covering values were highly variable (Fig. 7A), with a density positively correlated with the inclination of the substrate rather than the depth (Table 1, Fig. 8). The highest presence was found on vertical surfaces with covering values of 50 ± 13%. Covering values significantly reduced on horizontal surfaces to about 21 ± 6% at both depth ranges.

**Table 1:** Statistical Analysis.

Factor	df	Mean Sq	F value	Pr( > F)
Depth	1	40	0.372	0.549
Inclination	1	5017	46,609.000	1.23e-06***
Depth × Inclination	1	12	0.112	0.742
Residuals	20	108		

Two-way ANOVA, demonstrating main and interactive effects of Depth and Substrate Inclination on scleractinian species abundance (*n* = 24).

**Indicates significant effect (*p* < 0.01).

The quantitative samples’ analysis highlighted a mean coverage value of 37 ± 15% for *P. americana mouchezii*, and of 20 ± 15% for *P. muellerae*. However, *in situ* underwater observations led to local coverage values higher than 50% for this second species (Fig. 7B). Three additional scleractinian species, *Leptopsammia pruvoti* (Lacaze-Duthiers, 1897), *Caryophyllia inornata* (Duncan, 1878), and *Hoplangia durotrix* (Gosse, 1860), contributed to the reef construction, even with low coverage values. Living scleractinian colonies grew over a layer of dead corallites and were cemented by calcified polychaete tubes, which were organized into an in-place and interlocking meshwork that provided the reef rigidity.

Three species of serpulids, because of the large dimensions of their calcified tubes (up to 40–50 mm in length and 4–6 mm in diameter), contributed to the bioconstruction: *Serpula cavernicola* (Fassari and Mollica, 1991), *Hydroides pseudouncinata* Zibrowius, 1968 and *Janita fimbriata* (Delle Chiaje, 1822). Their abundances reached peaks greater than 150 tubes in 300 ml of sampled substrate (Fig. 7C). With strong tubes wrapped around themselves, the gregarious *Serpula massiliensis* also contributed to reef building. In addition, numerous specimens of *Vermiliopsis infundibulum* (Philippi, 1844) and *V. labiata* (O.G. Costa, 1861), with their strong and rather large tubes (20–30 mm in length and 2–3 mm in thickness), were detected on and around scleractinian corallites.

A vast number of individuals of the bivalve mollusc *Neopycnodonte cochlear* (Poli, 1795) formed aggregates on the top and inside the bioconstruction. Its abundance varied from 1 to 4 specimens in 300 ml of substrate (Fig. 7C). Thick layers of dead specimens were common inside the bioconstruction, where the valves were cemented to the dead corallites and the serpulid tubes contributed to the compactness of the structure (Fig. 6C–E). Other species of Mollusca, including the bivalves *Striarca lactea* (Linnaeus, 1758), *Hiatella rugosa* (Linnaeus, 1767), and *H. arctica* (Linnaeus, 1767), and the gastropod *Bittium latreillii* (Da Costa, 1778), added calcified material to the bioconstruction settling within interstices, surface hollows and crevices.

**Figure 9:**
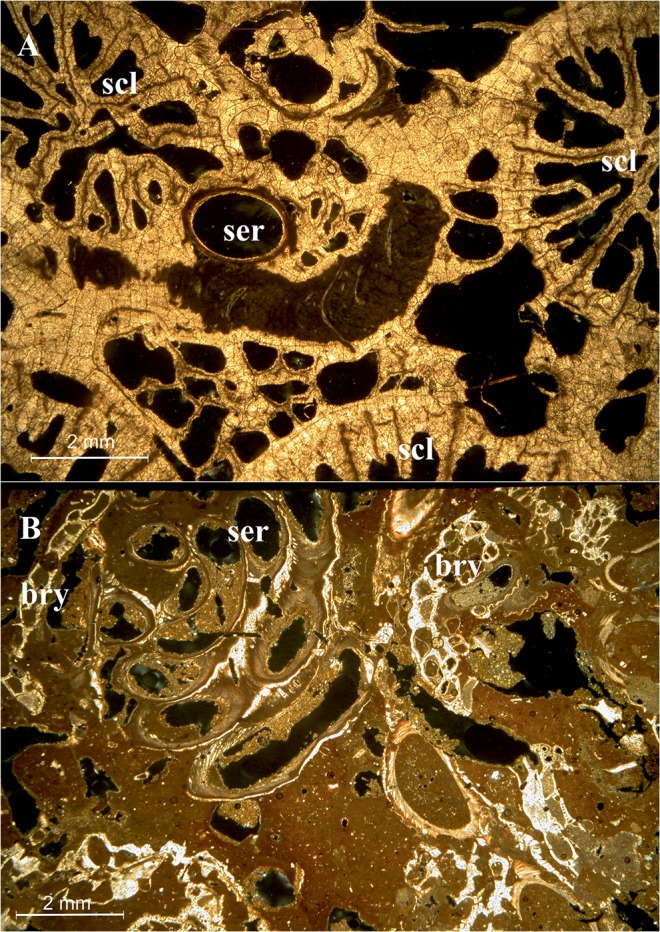
Plane-polarized light microscope photos of thin sections of the mesophotic coral reef. Details of the bioconstruction with (**A**) visible scleractinian corallites and a serpulid tube; (**B**) other carbonate-producing taxa, among which serpulids and bryozoans are recognizable (scl = scleractinian; ser = serpulid; bry = bryozoan).

Among bryozoans, the encrusting *Schizomavella* spp. were dominant on the external reef surface, with coverage values ranging from 1 to 15.8% (Fig. 7A). Less represented species included the erect *Myriapora truncata* (Pallas, 1766) and *Pentapora fascialis* (Pallas, 1766). Below the surface, builder scleractinian corallites were colonized by small encrusting colonies of the Cheilostomata *Puellina* (*Cribrilaria*) *radiata* and *Schizomavella cornuta* (=*auricolata*), together with colonies of *Beania magellanica* in the shape of crawling nets (Fig. 9). At a lower distributional limit of the MCR (a depth of approximately 30 m), building bryozoans replaced the scleractinians and assumed the role of the main constructors, with very extensive facies of *P. fascialis* intimately associated with the serpulid *Filograna* spp.

Red algae were represented by three Encrusting Coralline Rhodophyte species (ECR) that were only sporadically detectable (from 5.1 to 9.8%) during the analysis of both images and biological samples (Fig. 7A).

In addition to the reef-building species, few massive and erected demosponges (Porifera) were able to edify 3D substrates on the surface of the blocks (Figs 5, 6C), which contributed to the spatial heterogeneity of the reef. Analysis of the images indicated seven of these structuring species (*Aplysina cavernicola* (Vacelet, 1959); *Scalarispongia scalaris* (Schmidt, 1862); *Sarcotragus spinosulus* (Schmidt, 1862); *Petrosia ficiformis* (Poiret, 1789); *Haliclona mediterranea* (Griessinger, 1971); *Axinella cannabina* (Esper, 1794); and *Agelas oroides* (Schmidt, 1864)) were frequently detected with total covering values (all species combined) ranging from 1.7 to 67.5% (Fig. 7A). Additionally, the sponge assemblage was characterized by several endolithic forms. Among them, the most common species were the *Jaspis johnstonii* (Schmidt, 1862), *Dercitus* (*Stoeba*) *plicatus* (Schmidt, 1868) and *Triptolemma simplex* (Sarà, 1959), which play a role in linking the inner layer of the reef frame. The analysis of the biological samples indicated a few boring Demospongiae species (e.g., *Pione vastifica* (Hancock, 1849), *Cliona janitrix* (Topsent, 1932) and *C. schmidtii* (Ridley, 1881)) that widely infested carbonate concretions. In particular, *C. schmidtii* was observed in 100% of collected samples and in almost all scleractinian colonies. The bivalves *Lithophaga lithophaga* (Linnaeus, 1758) and *Rocellaria dubia* (Pennant, 1777) were also noted as bioeroder agents. Most of the sampled bivalve individuals belonged to the perforating species, which can create boring chambers in calcareous substrates; their valves formed holes and crevices among scleractinian corallites.

Finally, the non-building zoantharian *Parazoanthus axinellae* (Schmidt, 1862) was widely distributed, with covering values ranging from 1.7 to 40.2%. This taxon can form large facies that colonize scleractinian colonies and the external surface of several demosponges (Fig. 7A) (see Supplementary Videos 1, 2, 3, 4).

### Coral reef biodiversity

Within the considered taxonomic groups, the analysis of the biological samples revealed the presence of 153 taxa (Supplementary Table S3). The phylum Porifera had the greatest species richness, and was followed by Annelida, Polychaeta, Bryozoa, Mollusca, and Cnidaria, while algae were present but to a lesser extent.

### Algae

Nine algal taxa were detected in the present study. Four of them were identified to the genus level and five to the species level. Encrusting coralline algae and *Peyssonnelia* spp. were relatively uncommon. These groups mainly settled in areas with relatively low sediment deposition rates and patchy substrate coverage. All observed taxa belonged to the phylum Rhodophyta, which is divided into two classes: Florideophyceae, which was represented by eight taxa, and Stylonematophyceae, which was represented by only one taxon. In particular, only three encrusting coralline algae (non-geniculated) species of the class Florideophyceae were identified: *Lithophyllum stictaeforme* (Areshoug) Hauck (1878), *Neogoniolithon mamillosum* (Hauck) Setchell and L. R. Mason (1943) and *Titanoderma* spp. In addition, among structuring species, *Peyssonnelia inamoena* Pilger is a non-coralline crustose red alga that can make deep-water beds^34,35^.

### Porifera

The phylum Porifera was the most abundant taxonomic group, with a total of 59 taxa; of these, 57 were identified at the species level. The detected sponges belonged to the classes Homoscleromorpha and Demospongiae, with 2 and 57 species observed, respectively (Supplementary Table S3). Regarding Demospongiae, three subclasses (Verongimorpha, Keratosa and Heteroscleromorpha) were represented by 14 orders, 27 families and 40 genera. Three orders (Tetractinellida, Axinellida and Dictyoceratida) comprised approximately half of the sponge assemblages found (i.e., 28 taxa). Among them, the orders Tetractinellida and Dictyoceratida were the best represented and were divided into 3 families, 8 genera and 10 species and 4 families, 6 genera and 10 species, respectively. The orders Poecilosclerida and Suberitidae comprised 6 species each and were also well represented. The remaining orders were present with only a few species. The class Homoscleromorpha was represented by only one known order (Homosclerophorida), two families (Oscarellidae, Plakinidae) and two species.

### Cnidaria

**Figure 10:**
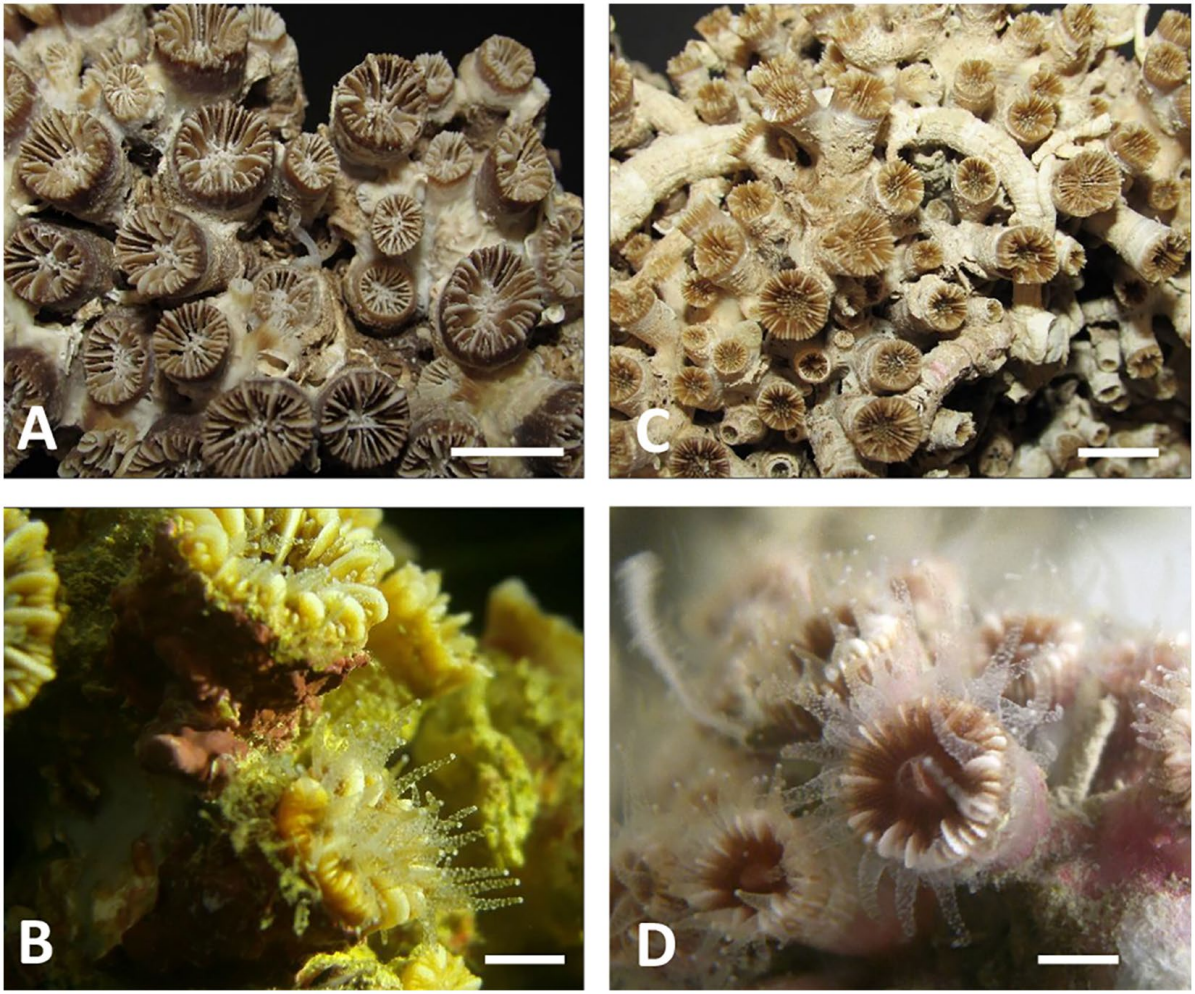
The main mesophotic coral reef contributors. Bleached and *in vivo* coral colonies of *Phyllangia americana mouchezii* (left column: **A**,**B**) and *Polycyathus muellerae* (right column: **C**,**D**). Scale bars: A, C = 1 cm; B, D = 0.5 cm.

A total of 10 species belonging to the classes Hydrozoa (1) and Anthozoa (9) were detected (Supplementary Table S3). Five Caryophylliidae and two Dendrophylliidae produced carbonate skeletons. Among them, two species, *Phyllangia americana mouchezii* and *Polycyathus muellerae*, were the principal reef builders. *P. americana mouchezii* generally formed cushion-shaped colonies that were as wide as 60 cm in diameter and coalesced in larger blocks (i.e., more than 100 cm in maximum length). The most widespread colonies were globose or hemispherical in shape, with subcylindrical corallites (sometimes slightly enlarged in the upper part) that were approximately 1.1 cm in diameter and up to 3.0 cm in height (Fig. 10A, B). They may give rise to lateral branches that grow parallel to the parent corallite. Polyps that were approximately 1.5 cm in diameter were apparently uncoloured. The histological observations did not reveal photosynthetic symbionts in the tissues (see Supplementary Fig. S1), in accordance with previous literature records^36,37^. *P. muellerae* developed irregular globular formations (up to 40 cm in diameter) that were characterized by little pinnacles and smaller subspherical agglomerates. These formations were tightly packed to form a continuum on wide areas of substrate. The corallites, which developed from a basal calcified matrix, were cylindrical in shape and characterized by a diameter ranging from 0.2 to 0.7 cm and a height of 1.5 cm (Fig. 10C, D). Their polyps had a diameter from 0.6 to 0.8 cm, were apparently uncoloured, and lacked photosynthetic symbionts (Supplementary Fig. S1), which is in agreement with histological observations and literature data^36,37^.

Other scleractinian species (e.g., *Caryophyllia* (*Caryophyllia*) *smithii*, *C*. (*C*.) *inornata*, *Hoplangia durotrix*, *Cladopsammia rolandi*, and *Leptopsammia pruvoti*) frequently occurred in the bioconstruction and were embedded among the largest blocks of *P. americana mouchezii* and *P. muellerae*. In particular, *L. pruvoti* formed large aggregations on shaded rock facies, under overhangs and in crevices. *H. durotrix* colonies were very small, composed of 5–6 corallites that were 0.3–0.4 cm in diameter. These patterns have been observed in small clusters with other solitary corals (e.g., *C*. (*C*.) *smithii* and *C*. (*C*.) *inornata*).

### Annelida Polychaeta

Thirty-four Polychaeta taxa (31 identified to the species level and 3 to the genus level) were counted, and they were shared among 4 orders and 10 families; thus, the polychaetes group was ranked second in terms of diversity. The order Sabellida, represented by the only family Serpulidae, comprised 50% of the Polychaeta assemblage associated with the coral reef. The orders Eunicida and Phyllodocida had 3 families and 9 taxa and 4 families and 7 taxa, respectively, while the order Amphinomida consisted of 1 family and 1 species. The family Serpulidae was the most represented in terms of abundance; specifically, *Hydroides pseuduncinata* and *Serpula massiliensis* were the most common species.

### Mollusca

The mollusc fauna sampled from the MCR of Monopoli had 19 living taxa identified to the species level. The assemblage was distributed among the classes Gastropoda (42%) and Bivalvia (58%), with 8 and 11 species, respectively. Except for the families Trochidae, Cerithiidae and Hiatellidae, each characterized by two species, the remaining families contained only one species. The deep-sea oyster *Neopycnodonte cochlear*, together with the bivalves *Hiatella rugosa*, *H. arctica* and the gastropod *Bittium jadertinumi*, were the most abundant species. All recorded specimens of *N. cochlear* were small in size.

### Bryozoa

Bryozoans comprised the third group in terms of diversity. Twenty-two bryozoan taxa were counted: 3 of them were identified to the genus level and 19 to the species level. All identified taxa belonged to the order Cheilostomatida (class Gymnolaemata). A total of 18 families were found, each containing one to three species. Among them, the families Celleporidae (containing three species), Microporidae and Bitectiporidae (each with two species) were the most represented. *Schizomavella cornuta* and *Arthropoma cecilii* were the most common species with their plurilaminar and unilaminar encrusting colonies. Additionally, *Puellina* (*Cribrilaria*) *radiata* and *Turbicellepora coronopus* were attached to the substrate with encrusting and celleporiform colonies, while the reticulate colonies of *Beania magellanica* were loosely adherent to the bioconstruction. With the exception of a few encrusting and erect species (e.g., *Schizomavella* spp., *Pentapora fascialis*, *Myriapora truncata*, *Adeonella calveti*, and *C. papyrea*), the detected taxa consisted mainly of small or thin encrusting specimens that were not visible to the naked eye (Fig. 9).

## Discussion

### The mesophotic coral reef

Although mesophotic coral ecosystems are widespread and diverse and their investigation is quickly increasing, they are still little explored in most part of the world^38^. Currently, in Mediterranean mesophotic environments biogenic formations are described as mainly dominated by octocorals, bryozoans or sponges^39–43^. Here we describe, for the first time in Mediterranean Sea, a MCR dominated by hexacorals, adding another piece to the marine bioconstruction puzzle knowledge.

The main contributors to reef building are two scleractinian species: *Phyllangia americana mouchezii* and *Polycyathus muellerae*. The outcrops show a remarkable thickness, up to 2 m, with an upper layer made by living builders that lean on the eroded skeletal rests of dead individuals and are cohesively maintained by numerous carbonatic polychaete tubes of different sizes (e.g., *Serpula cavernicola*, *S. massiliensis*, *Hydroides pseudouncinata*, *Janita fimbriata*, and *Vermiliopsis infundibulum*). Other invertebrates, such as the bivalve *Neopycnodonte cochlear*, poriferans and bryozoans, contribute to the high structural complexity and heterogeneity of the substrate.

Although the number of zoobenthic engineers is lower than that in tropical reefs^44^, these builder species are able to edify a spatially complex 3D framework of blocks characterized by a large number of holes and cavities with different microhabitats, hosting large amounts of associated species, thus showing the main traits of a coral reef ^9,10^.

In Mediterranean Sea, *Cladocora caespitosa* is considered the only bioconstructor scleractinian that hosts symbiotic zooxanthellae as tropical reef-building corals^45^. This species is part of the recent fossil history of the Mediterranean^15,16^, but it is currently patchily distributed and restricted to shallow waters^36^. On the other hand, the Mediterranean is also home to deep carbonate bioconstructions^17^ built by the so-called cold-water corals (CWCs), and particularly by the species *Desmophyllum pertusum* and *Madrepora oculata*, which do not host symbiotic dinoflagellates. The present study indicates that other scleractinian species lacking zooxanthellae are able to build large and well-structured bioconstructions showing the morphological traits of a coral reef.

In the literature, *P. americana mouchezii* and *P. muellerae* are reported to form laminar or cushion-shaped colonies, typically associated with dark or semi-dark habitats, but they are never described as reef-forming species^36,37^. Furthermore, both species are described as devoid of symbiotic dinoflagellates, as confirmed by the results of our histological observations (for more information, see Supplementary material). The described coral reef extends horizontally for approximately 2.5 km, in relation with the lateral extent of a reworked fault plane, over a relatively wide bathymetric range (depths of 30–55 m), and it covers a total of 0.05 km^2^. Its distribution is not continuous; rather, its development is mainly detectable on vertical/subvertical walls where living scleractinians have the highest covering values, while on horizontal surfaces the coral reef is locally interrupted by large lenses of incoherent sediments.

The extension of the coral reef does not seem to be limited to the investigated area but probably involves a much larger surface. Indeed, recent published images referred to the Apulian continental shelf^46^ suggest the occurrence of similar MCRs 100 km south from the area here investigated. In addition, widely extended bioconstructions built by *P. americana mouchezii*, *P. muellerae* and *N. cochlear* were recently found also on mesophotic bottoms about 50 km north from the study site (authors of the present manuscript, unpublished data).

The system described here could be widely distributed at Mediterranean scale, similarly to what described for the Red Sea, where scleractinians that lack symbionts play the role of main builder species in the wide carbonate structures that extend in mesophotic waters, up to depths of 200 m^47^. To date, only Mediterranean and Red Sea host peculiar MCRs produced by non-symbiotic scleractinians.

The occurrence of reefs built by scleractinians without the contribution of symbionts leads to the consideration that heterotrophy must necessarily play a considerable role in the metabolic processes of such species, supporting not only the basal metabolism but also the deposition of large amounts of calcium carbonate. Since feeding enhances the calcification rates in scleractinians associated with endosymbiotic dinoflagellates through various direct and indirect mechanisms^48–50^, it is reasonable to hypothesize that it has a similar role in species lacking symbionts. It is well known that the deep-water coral *D. pertusum* uses marine snow and detrital matter brought by deep currents as its main trophic resources^51,52^, forming coral bioconstructions (CWCs) where there is a continuous and regular supply of concentrated food and nutrients due to the deep current flow^53^. The MCR described here developed in an area affected by intermediate and deep currents that originate from northern Adriatic Sea and contain a large amount of nutrients mainly brought by the Po River^54,55^. Such currents flow to the south along Italian coasts and reach the Apulian shelf providing nourishment to the studied reef. In addition, the reef is located in the upper part of a continuous EW oriented morphostructural high (Fig. 3) that crosses the S-directed intermediate and has deep currents that induce local upwelling processes. The occurrence of many suspension feeder species associated with the coral reef further confirms the high trophic support of the surrounding waters.

Therefore, the present data constitute further evidence of the reef-building role of scleractinians without endosymbiotic dinoflagellates, stressing the role of trophic support of the surrounding waters and suggesting that the traditional ecological scenario in which coral reefs are associated with symbiotic processes between dinoflagellates and corals in oligotrophic waters should be reconsidered.

### Reef biodiversity

Among structuring benthic taxa, sponges were the richest and most diversified taxa. Their number (59 taxa) was higher than values reported in the literature in reference to the Apulian coast at a comparable depth^28^. Three species (*Suberites syringella* (Schmidt, 1868), *Raspaciona calva* (Sarà, 1958), and *Phorbas fibulatus* (Topsent, 1893) were new findings for the Italian Adriatic coast; twelve, corresponding to almost 22% of the total sponge assemblage detected, were endemic to the Mediterranean. Demosponges can play a triple role within the coral reef, acting as constructors that agglomerate carbonate particles, makers of 3D habitat (due to the occurrence of a large amount of massive/erect species) and eroders^41,56,57^, which mostly contribute to the dynamic equilibrium between growth and erosion phases of the bioconstruction^57^. Boring sponges, although here represented by few species (e.g., *Pione vastifica*, *Cliona janitrix* and *C. schmidtii*) were always present on coral skeletons, even though other calcareous substrates, such as the coiled and folded tubes of the polychaetes *S. cavernicola* and *S. massiliensis*, appeared lacking in erosion scars. These observations are consistent both with differences in skeleton micro-texture among taxa^58^ and with the specificity of the boring sponges bioerosion patterns^59^.

Annelid polychaetes were also widely represented, and among them Serpulidae was the most common group, frequently present with a large number of individuals. They are typically associated with the coralligenous community and less frequently with shallow bottoms, where they thrive in cryptic habitats within concretions^60,61^ and submarine caves^62^. Some of them also colonize deep-circalittoral down to bathyal bottoms and are already known to live on deep scleractinians in Mediterranean Sea^61,63^. *Vermiliopsis monodiscus* seems to be an endemic Mediterranean species, although it is considered rare in the Levantine basin^64,65^. In general, Serpulidae were represented by species with a wide ecological distribution, such as *Spirobranchus triqueter*, *Serpula vermicularis*, and *S. concharum*, but also by those typically associated with deep and dark environments (e.g., *V. monodiscus*, *Janita fimbriata*, and *Metavermilia multicristata*). *Semivermilia crenata*, *Placostegus crystallinus*, *Filogranula gracilis*, and *Vermiliopsis labiata* are reported in the literature as species typically associated with Mediterranean coralligenous concretions^60^. The largest recorded species was *Serpula cavernicola* Fassari and Mollica, 1991, which is usually found in submerged marine caves in Ionian and southern Tyrrhenian Seas. Among the vagile fauna, special mention must be given to the eunicid *Leodice torquata*, which had several large specimens that were recorded associated with corals living among scleractinian corallites. *L. torquata* is a carnivorous species that is probably linked to the presence of scleractinians, as observed in CWC banks, where *Eunice norvegica* is able to establish a symbiotic association with the dominant coral species *L. pertusa* and *M. oculata*^66^. This eunicid feeds on particles captured by the coral polyps and exhibits mutualistic behaviours cleaning the coral surface, attacking hazardous mobile organisms^67^, or aggregating the coral fragments to increase the strength of the bioconstruction^68^. The nature of this association in the MCR should be better investigated in order to define such species as opportunistic “nestlers” or true “bioeroders”.

The contribution of bryozoans to coverage was surprisingly low, considering that this group is frequently considered as one of the most important among invertebrate coralligenous builders^19,27^. They were observed over the entire bathymetric range of the reef, with small-size encrusting colonies mainly localized in the inner portions of the coral reef. Their abundance strongly increased with decreasing depths, where scleractinian corals were replaced by extensive facies of *Pentapora fascialis*.

The algal component was scarcely represented (9 taxa), with only three species of coralline algae displaying low values of substrate coverage. All the detected species have been already reported in deep-water environments^34,69^. However, the absence of most of the sciaphilous macroalgae usually associated with deep Mediterranean coralligenous assemblages, together with the occurrence of only few Rhodophyta species, is a circumstance that remains unexplained. This result is very peculiar because coralligenous algae are known for their ability to survive in deep waters where luminosity is very low, and some species have been found at depths greater than 250 m^70^. Sedimentation is one of the limiting factors for their growth, together with acidification^71,72^, grazing^73^ and turf coverage^74^. Indeed, in the studied area, the high sedimentation rate could be the main limiting factor for the growth of algae, as suggested by the low number of algal species and the occurrence of only non-geniculated species^75^.

Overall, from a taxonomic point of view, the MCR benthic assemblage showed a marked similarity with Mediterranean coralligenous communities. Indeed, more than 80% of the species censused here have been previously reported in the literature in relation to coralligenous communities, with large variations among different taxa (Cnidaria, 100%; Porifera, 88%; Bryozoa, 70%; Mollusca, 89%; Polychaeta, 79%)^19,42,56,59,76–79^.

Coralligenous is a very heterogeneous habitat where different organisms can contribute to the bioconstruction (i.e. from calcareous red algae to invertebrates), according to the environmental features^19^. Along the Apulian coast, coralligenous is well represented and diversified, with 97 algal and 611 benthic invertebrate species censused in total^80^. Although the bioconstruction described here is contiguous and taxonomically similar to coralligenous communities, it appears consistently different from a structural point of view, being mainly built by scleractinians, organized into an in-place and interlocking meshwork that provides rigidity to the reef, together with some auxiliary engineering species (e.g., *N. cochlear*), while the contribution of the algal component appears negligible. For these reasons, this bioconstruction deserves in all respects the definition of “coral reef”. Therefore, we propose to categorize the MCR here described and its highly-diversified community as a further bioconstruction that contributes, together with the typical coralligenous and CWC bioconstructions, to the main heterogeneous typologies of Mediterranean bioconstructions.

## Methods

Different methodologies were applied to produce a geomorphological and bionomic map of the study area, analyse the taxonomic and structural composition of the bioconstruction, and describe its structural organization. The study envisaged the creation of a morpho-bathymetric map using geophysical acquisition techniques. The resulting data were then used for the establishment of an informative level of the bionomic features of the investigated area. The obtained bionomic map was validated through remotely operated vehicle (ROV) transects and underwater diving. The main facies present in the area were identified and successively codified according to the BIOMAP (marine bioconstructions in Apulia) protocol^80^. Once the area of interest (dominated by scleractinian corals) had been defined in terms of occupied surface area and bathymetric extension, further dives were conducted to collect the images and biological samples used for subsequent laboratory analyses.

### Study area

The study area was located in the northern sector of Monopoli, along the southern Adriatic coast of Italy (Fig. 1). This sector of the Apulian foreland shows relatively simple geological features, with a WNW-ESE-trending ridge mainly composed of Mesozoic limestones. This ridge appears segmented by large E-W oriented fault zones^81^. In the eastern flank of the Murge area, the Calcarenite di Gravina Formation records the subsidence phase that involves the entire Apulian Foreland during the late Pliocene-early Pleistocene. Within the area of Monopoli, this formation transgressively overlies the upper Cretaceous limestones of the Calcare di Bari Formation. The unconformity is located approximately at sea level^82,83^. This rocky coastal area contains some ephemeral streams that carry moderate amounts of sediments to the sea during severe weather events. A microtidal setting characterizes this sector of the Mediterranean Sea, and the coastal dynamics are wave-dominated. The wave conditions (1968–2008 data via a wave buoy at Monopoli - N40°58′30.0″, E17°22′36.1″) indicate that the prevailing direction of sea storms is from the northwest, and the main coastal longshore currents occur in the NW-SE direction. Here, sediments in shallow marine environments are made up of terrigenous material: siliciclastics, carbonate lithoclasts and variable contents of bioclasts^84,85^. The submerged morphology is modelled in a rocky and soft-sediment substrate and is dominated by the following: linear features that are perpendicular to the present-day coastline (they are the continuation of main streams); parallel features that related to the interaction between the low-rate regional uplift and the eustatic sea-level changes (they are erosional/depositional in origin); and transverse EW-oriented features that are probably tectonic in origin. In this area, from a biological point of view, the literature data report a succession of biocoenosis from photophilous to sciaphilous hard substrate up to 55 m of depth, beyond which occurs a bottom of pelitic sand^80^. The coastal area is characterized by shallow photophilous communities with encrusting calcareous algae, followed by a biocoenosis that consists of rocky areas covered by calcareous algae that alternate with sandy lenses occupied by *Posidonia oceanica*. In the bathymetric range encompassed between 10 and 20 m, the soft bottoms are covered by *Cymodocea nodosa* meadows. Coralligenous constructions increase with increasing depths. After exceeding 20 m of depth, numerous bottom jumps with the appearance of coralligenous wall formations can be seen. The maximum development is represented by a biocoenosis of platform coralligenous, whose massive isolated formations rise from the incoherent bottom, reaching a height from 4 to 6 m. At approximately 55 m of depth, the bottom is composed of muddy fine sediments with rare rocky outcrops.

### Geomorphological and bionomic maps

The morpho-bathymetric map with an area of 4.8 km^2^ was obtained by using a CHIRP pulse side-scan sonar (SSS) (manufactured by BENTHOS - USA model SIS1500, FM 190–210 kHz) coupled with the sub-bottom profiler (SBP) (manufactured by BENTHOS - USA model Chirp II double operational frequencies 2 kHz–7 kHz). Data collected with SSS and SBP in May 2017, were processed by means of the software CARIS SIPS and IXSEA Delph (France/UK Seismic data processing suite), respectively. The navigation was obtained from a differential global positioning system (DGPS) GPS (manufactured by TRIMBLE - USA/UK model SPS551) by means of the navigation software RESON PDS2000. All processed data were inserted in a geographic information system project (GIS ESRI ArcView 10.2; projection UTM33N-WGS84). The bathymetric information collected from different sources and acquired in past surveys by other geophysical teams were checked and merged to produce a final unique bathymetric dataset and input in the GIS environment as per acquired and processed data. Seabed classification was based on SSS and SBP data analysis and on mosaicked geo-referenced images produced at the end of the processing phase. A high-resolution grey-scale image was analysed, mapped and cross-correlated to SBP data. In detail, both the raw dataset and the processed mosaicked images were accurately analysed and accordingly classified. In the GIS environment, detailed mapping was executed on geo-referenced images as a polygon layer for a complete coverage of the surveyed area. Seabed characteristics were identified based on the geophysical features detected from the collected dataset. In detail, the SSS images allowed for the clear identification of the main seabed types. Moreover, the cross correlation of this information with the SBP dataset, in terms of seismic signal penetration and scattering below seabed geometrical evidence in detected sedimentary sequences, led to the improvement of the seven seabed-type classifications, as reported in Supplementary Table S1. These categories were visually defined by the dominant biological structure, measured by a combination of the size and density of the biogenic structures present and used to define the further analysis protocol (Supplementary Table S2).

### Video acquisition and sample collection

To validate the interpretation of the mosaicked sonograms, describe the “architecture” of the reef and characterize the associated epibenthic assemblages in terms of structuring taxa, 11 ROV (Mariscope FO III) transects and 6 scuba-diving observations were performed (Fig. 2) in August and September 2017. ROV profiles and scuba diving were selected according to the bionomic map, in correspondence with passages from one facies to another or where the signal that was returned from geophysics was not sufficient to exactly define the type of biological association that was present (e.g., coralligenous *sensu strictu* or coral reef). The Marine Strategy Framework Directive (MSFD) protocols were followed for the ROV transects and scuba diving. The ROV was equipped with high-definition video cameras, high-performance LED illuminators, a HERO4 GoPro, and an underwater acoustic tracking position system (Applied Acoustic Smart Track), which provided records of its track along the seabed. Additionally, three laser beams at a distance of 10 cm from each other projected on the substrate, approximately in the centre of the image, and were used for the extraction of quantitative data (e.g., body size, covering estimations). The navigation software RESON PDS2000 continuously recorded the position of both the ROV and the ship to geo-reference the images on seabed.

Ten samples, each of approximately 3 L in volume, were collected in different areas and depth intervals of the coral reef by scuba divers for taxonomical analysis (Fig. 2: Dive 01, Dive 02, Dive 04, Dive 05, Dive 06). In addition, the largest (approximately 0.4 × 0.3 × 0.2 m) oriented and undisturbed samples were also collected from the same areas to describe the structural organization of the reef.

### Taxonomic and structural analysis

Sampled soft-bodied invertebrates were immediately transported to the laboratory, sorted and then anaesthetized with a saturated menthol solution in sea water. Later, the complete biological material was sorted, and all species were fixed in a 5% formaldehyde solution with seawater and stored in a 70% ethanol solution. To identify sampled taxa, an appropriate procedure of preparation and identification of each taxonomic category was applied. The collected biological material was identified to the lowest possible taxonomic level. The taxonomic nomenclature referred to the World Register of Marine Species (WORMS).

The distribution pattern of the structuring sessile taxa was evaluated by 120 snapshots extracted by means of the freely available DVDVideoSoft Free Studio software from video ROV transects and scuba dive videos recorded on the mesophotic coral reef (Fig. 2: Dive 01, Dive 02, Dive 04, Dive 05, Dive 06, ROV 01, ROV 02, ROV 04, ROV 05). A set of snapshots (*n* = 24) related to vertical and horizontal substrates and depth intervals (35–40 and 45–50 meters), were acquired, aiming to describe the species distribution pattern. For each image the covering percentage of scleractinian species, the inclination of the substrate (vertical/horizontal) and the depth was recorded. Coverage values were tested by two-way analysis of variance (ANOVA) on the hypothesis of significant differences between depth and substrate inclination.

The relative abundance of the scleractinians *Phyllangia americana mouchezii* and *Polycyathus muellerae* was calculated using the sampled material since taxonomic differences were not detectable with reliable precision by means of photographic images. Image analysis was performed using ImageJ software. Serpulid polychaete and mollusc contributions to the structure of bioconstruction were evaluated as the abundance of individuals present in three randomly selected 300 ml volume substrate units for each sample. For this purpose, serpulid individuals were divided into 3 size classes (I, II, III, referring to 1, 3 and 5 mm of tube diameter, respectively).

With the aim of describing the 3D structure of the outcrops, the large collected samples were washed in the laboratory with distilled water and dried in the stove. A complete impregnation with low viscosity epoxy resin was carried out to preserve the original bioconstruction framework during the cutting of slices with a circular saw. An impregnation procedure was specifically built for these samples and involved holding the samples in a plastic coat and a large vacuum bell. A minimum of six cycles of impregnation were needed for each sample. High-resolution images of large-scale slices were obtained with a scanner, while for the thin sections (30 µm), the camera of a petrographic microscope was used. Image analyses were carried out using ImageJ software to investigate the structure of the reef and the distribution of main builder taxa from the outer layer towards the inner layer of the bioconstruction and to evaluate their covering value in the section.

### Histological analysis

To verify the presence of photosynthetic symbionts, live corallites of the main builder scleractinian species were additionally sampled and then fixed for 3 hours in 4% paraformaldehyde solution in 0.1 M phosphate-buffered saline (PBS) at a pH of 7.4 and 4 °C. After rinsing in PBS, pieces were immersed in PBS with added 6.8% sucrose and then incubated overnight at 4 °C, in PBS with 6.8% added sucrose, and then dehydrated with increasing acetone at 4 °C. Samples were then subjected to the infiltration by incubation in a Technovit 8100 monomer (EMS, Hatfield, PA) for 6 hours at 4 °C. Finally, samples were embedded with an ice-cold 15:1 infiltrating solution. Polymerization was performed on an ice bed for 3 hours. Semi-thin sections (2 mm) were cut with glass knives using an LKB Ultratome and mounted on microscope slides, coated with polylysine and stained with toluidine blue to assess the general morphology of tissues^86^.

## Supplementary information

Supplementary file

Supplementary Video Mesophotic Coral Reef 1.

Supplementary Video Mesophotic Coral Reef 2.

Supplementary Video Mesophotic Coral Reef 3.

Supplementary Video Mesophotic Coral Reef 4.

## Data Availability

All data generated or analysed during this study are included in this published article and its Supplementary Information files.
