# Supplementary material for: A Mediterranean mesophotic coral reef built by non-symbiotic scleractinians
Source: Sci Rep. 2019 Mar 5;9:3601. doi: 10.1038/s41598-019-40284-4 (PMC6401148; doi:10.1038/s41598-019-40284-4)

Supplementary Information for

A Mediterranean mesophotic coral reef built by non-symbiotic scleractinians

Giuseppe Corriero, Cataldo Pierri, Maria Mercurio, Carlotta Nonnis Marzano, Senem Onen Tarantini, Maria Flavia Gravina, Stefania Lisco, Massimo Moretti, Francesco De Giosa, Eliana Valenzano, Adriana Giangrande, Maria Mastrodonato, Caterina Longo, Frine Cardone

SUPPLEMENTARY TABLE AND FIGURE CAPTIONS

Table S1. Seabed classes related to SSS and SBP sample images from original dataset.

Table S2. Benthic bionomy of the study area.

Table S3. List of the benthic taxa recorded in the Monopoli mesophotic coral reef with bathymetric range (in meter) occurrence.

Figure S1. Light microscopy of the polyps of the main mesophotic coral reef contributors. A: *Phyllangia americana mouchezii*; B: *Polycyathus muellerae*. Epidermis (ep), gastrodermis (gs), mesoglea (ms), mucocytes (mc), and nematocyst (stinging cell) (sc). Scale bars = 20 μ.

SUPPLEMENTARY VIDEO CAPTIONS

These videos were recorded by scuba divers on the Monopoli mesophotic coral reef (MCR) at a depth ranging from 40 to 45 m.

Supplementary Video Mesophotic Coral Reef 1.
Video recorded on a sub-horizontal slope. In this video are well visible the cushion-shaped bioconstruction mainly erected by the scleractinian *Phyllangia americana mouchezii* colonized by the orange zoantharian *Parazoanthus axinellae*, various sponges such as *Petrosia ficiformis*, massive Keratosa, the erect *Axinella cannabina* and other incrusting taxa. Are also visible encrusting plaques of the bryozoan *Schizomavella* spp..

Supplementary Video Mesophotic Coral Reef 2.
Video recorded on a sub-vertical slope. In this video is evident the heavy siltation over the reef structure. Here is easy recognizable the structural complexity of the bioconstructions and their marked thickness development. The orange zoantharian *Parazooanthus axinellae* and various sponges (the erect *Axinella cannabina*, massive Keratosa, *Aplysina cavernicola, Haliclona mediterranea* and other encrusting taxa) mainly colonize the coral blocks. Are also visible rare calcareous encrusting red algae.

Supplementary Video Mesophotic Coral Reef 3.
This video shows a detail of the Monopoli MCR in which are recognizable the globose coral blocks, *Parazoanthus axinellae*, sponges (*Axinella cannabina,* Keratosa, *Aplysina cavernicola,* other encrusting taxa) and the encrusting bryozoan *Schizomavella* spp..

Supplementary Video Mesophotic Coral Reef 4.
This video shows a detail of the Monopoli MCR characterized by one of the structuring sponge species *Aplysina cavernicola*. Well recognizable are also the erected sponges *Axinella cannabina* and massive Keratosa.

Table S1. Seabed classes related to SSS and SBP sample image from original dataset.

Classes	SSS mosaic sample image	SBP sample image	Geophysical features description
Coralligenous *sensu strictu*	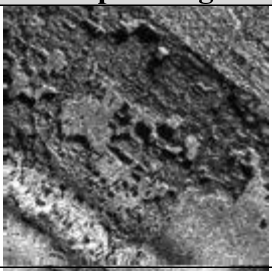	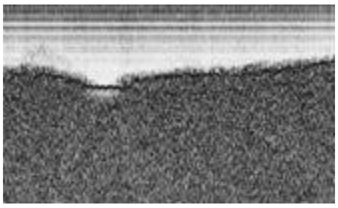	Rugged high reflectivity seabed 1.0–1.5 m raised on close loose sediment areas. No SBP seismic signal penetration below these features detected.
Mosaic of coralligenous outcrops and coastal detritic	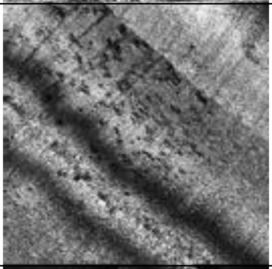	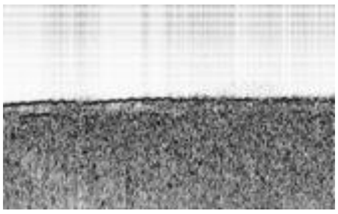	Scattered objects 1.0–1.5 m high spread on medium-low reflectivity smooth seabed. Scattered SBP seismic signal penetration in those areas.
Mesophotic coral reef	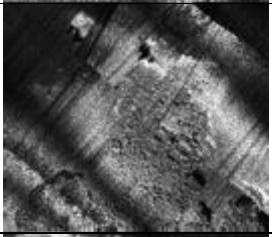	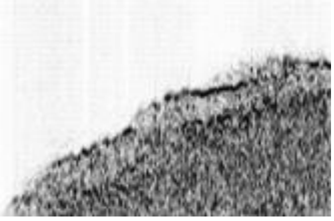	Compact and rugged very high reflectivity seabed at top and on flank of seabed slope. No SBP seismic signal penetration below those features detected.
Mesophotic coral reef patches on fine soft sediments	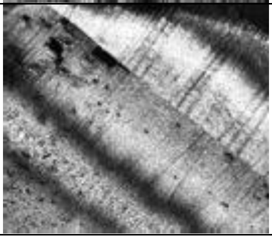	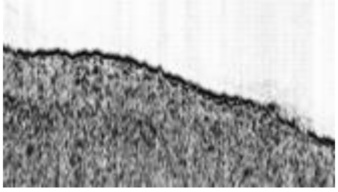	Scattered objects 1.0–1.5 m high spread on high reflectivity smooth seabed areas. Scattered SBP seismic signal penetration in those areas.
Coarse loose sediments	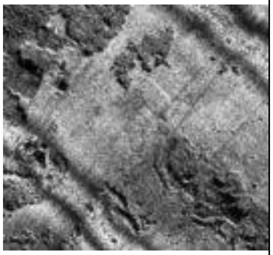	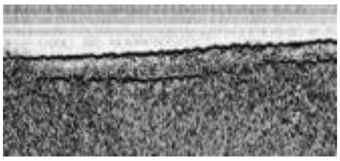	Medium-high reflectivity smooth seabed mainly occurring in glades and in erosional grooves in high reflectivity smooth or lightly rugged seabed in coastal areas. SBP seismic signal penetration occurs in areas showing likely loose sediment layers with up to 4 m thickness under seabed.
Rocky seabed	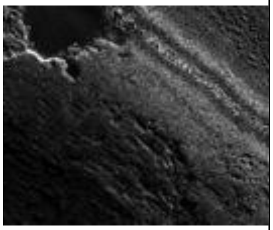	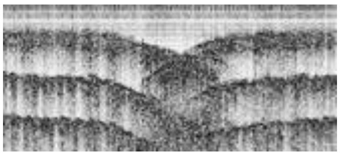	High reflectivity smooth or lightly rugged seabed occurring in coastal areas clearly marked by erosional grooves ('lame') having SW-NE direction often filled by coarse loose sediments. No SBP seismic signal penetration below those features detected.

Fine soft sediments	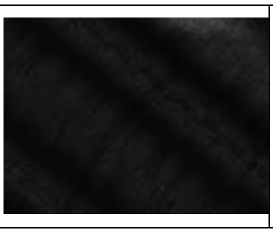	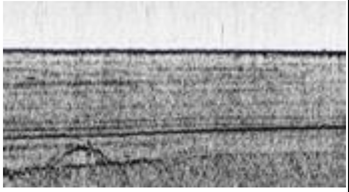	Low reflectivity smooth seabed occurring in distal areas (>30 m w.d.). SBP seismic signal penetration shows sediments series thickness up to 12 m below seabed.

Table S2. Benthic bionomy of the study area.

Classes	Description
Coralligenous	Bioconstruction mainly made by coralline algae dominated by erect, foliaceous or encrusting forms associated with invertebrate suspension feeders such us sponges, bryozoans, gorgonians and scleractinian corals
Mosaic of coralligenous and coastal detritic	Mosaic of coralligenous bioconstructions mixed with coarse terrigenous and organogenous incoherent substrate
Mesophotic coral reef	Bioconstruction mainly made by scleractinian corals associated with sponges, mollusks and bryozoans
Mesophotic coral reef patches and fine sediments	Mesophotic coral reef patches and fine (sandy clay/silt) soft sediments
Biocoenosis of infralittoral detritic bottom	Coarse sand, fine gravel occurring in littoral areas and erosional lineations (lame) with or without vegetal coverage
Biocoenosis of infralittoral algae	Biocoenosis of infralittoral photophilous algae dominated by frondose coralline and tiny brown algae

Table S3. List of the benthic taxa recorded in the Monopoli mesophotic coral reef with bathymetric range (in meter) occurrence.

Phylum	Class	Family	Species	Depth (m)
RHODOPHYTA	FLORIDEOPHYCEAE	Corallinaceae	*Lithophyllum stictaeforme* (J.E. Areschoug) Hauck, 1877	35-45
			Neogoniolithon mamillosum (Hauck) Setchell & L.R.Mason, 1943	30-45
			Titanoderma sp.	45
		Peyssonneliaceae	*Peyssonnelia inamoena* Pilger, 1911	30-45
		Rhodymeniaceae	*Rhodymenia* sp.	45
		Ceramiaceae	*Ceramium* sp.	45
			Antithamnion tenuissimum (Hauck) Schiffner, 1916	30-35
		Delesseriaceae	*Hypoglossum* sp.	35
	STYLONEMATOPHYCEAE	Stylonemataceae	*Stylonema alsidii* (Zanardini) K.M.Drew, 1956	45
PORIFERA	HOMOSCLEROMORPHA	Plakinidae	*Plakortis simplex* Schulze, 1880	35-45
		Oscarellidae	*Oscarella lobularis* (Schmidt, 1862)	35-40
	DEMOSPONGIAE	Ancorinidae	*Stelletta stellata* Topsent, 1893	35-45
			Jaspis johnstonii (Schmidt, 1862)	35-45
			Stryphnus ponderosus (Bowerbank, 1866)	45-45
			Holoxea furtiva Topsent, 1892	45
			Dercitus (Stoeba) dissimilis (Sarà, 1959)	35-45
			Dercitus (Stoeba) plicatus(Schmidt, 1868)	35-45
		Geodiidae	*Erylus discophorus* (Schmidt, 1862)	45
			Geodia conchilega Schmidt, 1862	40-45
			Geodia sp.	45
		Pachastrellidae	*Triptolemma simplex* (Sarà, 1959)	35-45
		Clionaidae	*Cliona janitrix* Topsent, 1932	40-45
			Cliona schmidti (Ridley, 1881)	40-45
			Pione vastifica (Hancock, 1849)	35-45
		Spirastrellidae	*Spirastrella cunctatrix* Schmidt, 1868	30-40
		Suberitidae	*Aaptos aaptos* (Schmidt, 1864)	40-45
			Protosuberites denhartogi van Soest & de Kluijver, 2003	35-45
			Suberites carnosus (Johnston, 1842)	35-45
			Suberites massa Nardo, 1847	45
			Suberites syringella (Schmidt, 1868)	45
			Terpios gelatinosus (Bowerbank, 1866)	35-45
		Hymerhabdiidae	*Prosuberites longispinus* Topsent, 1893	35-45
		Tethyidae	*Tethya* cf. *citrina* Sarà & Melone, 1965	45
		Chondrosiidae	*Chondrosia reniformis* Nardo, 1847	35-40
		Raspailiidae	*Eurypon cinctum* Sarà, 1960	45
			Eurypon viride (Topsent, 1889)	45
			Raspaciona aculeata (Johnston, 1842)	35-45
			Raspaciona calva Sarà, 1958	45
		Coelosphaeridae	*Lissodendoryx (Anomodoryx) cavernosa* (Topsent, 1892)	45
		Hymedesmiidae	*Phorbas fibulatus* (Topsent, 1893)	40-45
			Phorbas fictitius (Bowerbank, 1866)	30-40
			Phorbas tenacior (Topsent, 1925)	30-45
		Biemnidae	*Biemna parthenopea* Pulitzer-Finali, 1978	45
		Mycalidae	*Mycale (Aegogropila) tunicata* (Schmidt, 1862)	45
			Mycale (Aegogropila) contarenii (Lieberkühn, 1859)	30-40
		Axinellidae	*Axinella cannabina* (Esper, 1794)	35-55
			Axinella damicornis (Esper, 1794)	35-40
			Axinella polypoides Schmidt, 1862	45
			Axinella verrucosa (Esper, 1794)	35-50
		Bubaridae	*Bubaris vermiculata* (Bowerbank, 1866)	45
		Dictyonellidae	*Acanthella acuta* Schmidt, 1862	35-40
		Agelasidae	*Agelas oroides* (Schmidt, 1864)	35-50
		Chalinidae	*Haliclona (Reniera) mediterranea* Griessinger, 1971	35-50
			Haliclona (Haliclona) simulans (Johnston, 1842)	40-50

		**Petrosiidae**	*Petrosia (Petrosia) ficiformis* (Poiret, 1789)	30-55
		**Irciniidae**	*Ircinia variabilis* (Schmidt, 1862)	40-50
			Ircinia oros (Schmidt, 1864)	45-50
			Sarcotragus foetidus (Schmidt, 1862)	35 -50
			Sarcotragus spinosulus Schmidt, 1862	35 -50
		**Thorectidae**	*Scalarispongia scalaris* (Schmidt, 1862)	35-50
			Fasciospongia cavernosa (Schmidt, 1862)	45
		**Spongiidae**	*Spongia (Spongia) officinalis* Linnaeus, 1759	45-50
		**Dysideidae**	*Dysidea fragilis* (Montagu, 1818)	45-50
			Dysidea sp.	40-50
			Pleraplysilla spinifera (Schulze, 1878)	35-50
		**Dictyodendrillidae**	*Spongionella depressa* Topsent, 1929	40-45
		**Aplysinidae**	*Aplysina cavernicola* Vacelet, 1959	40-55
		**Ianthellidae**	*Hexadella racovitzai* Topsent, 1896	40-45
**CNIDARIA**	**HYDROZOA**	**Eudendriidae**	*Eudendrium ramosum* (Linnaeus, 1758)	35-40
	**ANTHOZOA**	**Gorgoniidae**	*Leptogorgia sarmentosa* (Esper, 1789)	40
		**Caryophylliidae**	*Caryophyllia smithii* Stokes & Broderip, 1828	40-45
			Caryophyllia inornata (Duncan, 1878)	30-45
			Hoplangia durotrix Gosse, 1860	35-45
			Phyllangia americana mouchezii (Lacaze-Duthiers, 1897)	30-55
			Polycyathus muellerae (Abel, 1959)	30-55
		**Dendrophylliidae**	*Cladopsammia rolandi* Lacaze-Duthiers, 1897	45
			Leptopsammia pruvoti Lacaze-Duthiers, 1897	40-50
		**Parazoanthidae**	*Parazoanthus axinellae* (Schmidt, 1862)	35-55
**MOLLUSCA**	**GASTROPODA**	**Fissurellidae**	*Emarginula octaviana* Coen, 1939	35-45
		**Trochidae**	*Clanculus corallinus* (Gmelin, 1791)	40-45
			Clanculus crociatus (Linnaeus, 1758)	40-45
		**Cerithidae**	*Bittium reticulatum* (da Costa, 1778)	35-45
			Bittium latreillii (Payraudeau, 1826)	30-45
		**Turritellidae**	*Turritella communis* Risso, 1826	45
		**Triphoridae**	*Marshallora adversa* (Montagu, 1803)	35-45
		**Muricidae**	*Muricopsis cristata* (Brocchi, 1814)	45
	**BIVALVIA**	**Arcidae**	*Barbatia barbata* (Linnaeus, 1758)	35-45
		**Noetiidae**	*Striarca lactea* (Linnaeus, 1758)	40-45
		**Mytilidae**	*Lithophaga lithophaga* (Linnaeus, 1758)	35-45
		**Anomiidae**	*Pododesmus patelliformis* (Linnaeus, 1761)	45
		**Gryphaeidae**	*Neopycnodonte cochlear* (Poli, 1795)	40-55
		**Chamidae**	*Chama gryphoides* Linnaeus, 1758	35-45
		**Kelliidae**	*Kellia suborbicularis* (Montagu, 1803)	40-45
		**Trapezidae**	*Coralliophaga lithophagella* (Lamarck, 1819)	50
		**Gastrochaenidae**	*Rocellaria dubia* (Pennant, 1777)	30-45
		**Hiatellidae**	*Hiatella rugosa* (Linnaeus, 1767)	35-45
			Hiatella arctica (Linnaeus, 1767)	35-45
**ANNELIDA**	**POLYCHAETA**	**Polynoidae**	*Lepidasthenia elegans* (Grube, 1840)	40-45
		**Syllidae**	*Syllis* spp.	30-45
		**Nereididae**	*Ceratonereis hircinicola* (Eisig, 1870)	35-45
		**Glyceridae**	*Glycera tesselata* Grube, 1863	30-45
			Glycera unicornis Lamarck, 1818	30-45
			Glycinde nordmanni (Malmgren, 1866)	35-40
		**Goniadidae**	*Goniada maculata* Örsted, 1843	40-45
		**Eunicidae**	*Leodice torquata* (Quatrefages, 1866)	35-45
			Lysidice collaris Grube, 1870	30-45
			Lysidice ninetta Audouin & Milne-Edwards, 1833	30-40
			Lysidice unicornis (Grube, 1840)	40-45
			Lysidice sp.	30-45
			Marphysa sp.	35-45
			Palola siciliensis (Grube, 1840)	35-45
		**Lumbrineridae**	*Scoletoma impatiens* (Claparède, 1868)	45
		**Oenonidae**	*Arabella geniculata* (Claparède, 1868)	35-45

		**Euphrosinidae**	*Euphrosine foliosa* Audouin & Milne-Edwards, 1833	30-45
		**Sabellidae**	*Hypsicomus stichophthalmos* (Grube, 1863)	35-45
		**Serpulidae**	*Hydroides pseudouncinata* Zibrowius, 1968	40-45
			Janita fimbriata (Delle Chiaje, 1822)	40-45
			Placostegus crystallinus sensu Zibrowius, 1968	40-45
			Filogranula gracilis Langerhans, 1884	30-40
			Metavermilia multicristata (Philippi, 1844)	40-45
			Semivermilia crenata (O.G. Costa, 1861)	40-45
			Serpula cavernicola Fassari & Mollica, 1991	40-50
			Serpula concharum Langerhans, 1880	40-50
			Serpula vermicularis Linnaeus, 1767	30-50
			Spiraserpula massiliensis (Zibrowius, 1968)	35-45
			Spirobranchus triqueter (Linnaeus, 1758)	40-45
			Vermiliopsis infundibulum (Philippi, 1844)	40-45
			Vermiliopsis labiata (O.G. Costa, 1861)	35-45
			Vermiliopsis monodiscus Zibrowius, 1968	30-40
			Vermiliopsis striaticeps (Grube, 1862)	30-40
			Filograna implexa Berkeley, 1835	30-50
**BRYOZOA**	**GYMNOLAEMATA**	**Flustridae**	*Chartella papyrea* (Pallas, 1766)	45
		**Beaniidae**	*Beania magellanica* (Busk, 1852)	40-45
		**Microporidae**	*Calpensia nobilis* (Esper, 1796)	45
			Mollia patellaria (Moll, 1803)	35-45
		**Cribrilinidae**	*Puellina radiata* (Moll, 1803)	40-45
		**Chorizoporidae**	*Chorizopora brongniartii* (Audouin, 1826)	30-45
		**Adeonellidae**	*Adeonella calveti* (Canu & Bassler, 1930)	45-55
		**Romancheinidae**	*Escharella variolosa* (Johnston, 1838)	35-45
		**Bitectiporidae**	*Pentapora fascialis* (Pallas, 1766)	35-40
		**Smittinidae**	*Smittoidea reticulata* (MacGillivray, 1842)	35-45
		**Bitectiporidae**	*Schizomavella (Schizomavella) cornuta* (Heller, 1867)	35-55
			Schizomavella (Schizomavella) mamillata (Hincks, 1880)	35-55
		**Schizoporellidae**	*Schizoporella* sp.	30-45
		**Myriaporidae**	*Myriapora truncata* (Pallas, 1766)	35-45
		**Microporellidae**	*Microporella ciliata* (Pallas, 1766)	30-45
		**Lacernidae**	*Arthropoma cecilii* (Audouin, 1826)	30-45
		**Celleporidae**	*Cellepora pumicosa* (Pallas, 1766)	40-45
			Celleporina caminata (Waters, 1879)	45
			Turbicellepora coronopus (Wood, 1844)	40-45
		**Phidoloporidae**	*Reteporella* spp.	40-55
			Rhynchozoon sp.	45
		**Hippaliosinidae**	*Hippaliosina depressa* (Busk, 1854)	40-45

Figure S1. Light microscopy of the polyps of the main mesophotic coral reef contributors. A: *Phyllangia americana mouchezii*; B: *Polycyathus muellerae*. Epidermis (ep), gastrodermis (gs), mesoglea (ms), mucocytes (mc), and nematocyst (stinging cell) (sc). Scale bars = 20 μ.

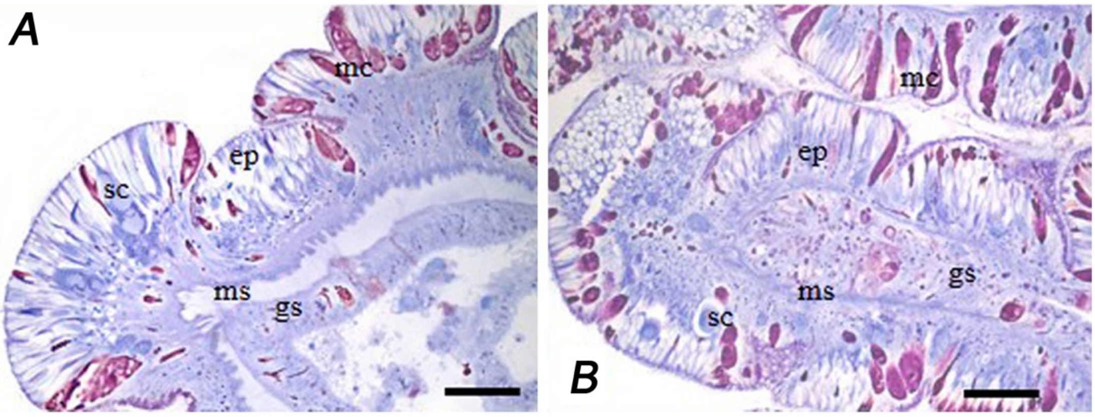

Supplement: Supplementary file 1 — Supplementary file [file 41598_2019_40284_MOESM1_ESM.pdf]
